# Identification of a Novel Adélie Penguin Circovirus at Cape Crozier (Ross Island, Antarctica)

**DOI:** 10.3390/v11121088

**Published:** 2019-11-22

**Authors:** Virginia Morandini, Katie M. Dugger, Grant Ballard, Megan Elrod, Annie Schmidt, Valeria Ruoppolo, Amélie Lescroël, Dennis Jongsomjit, Melanie Massaro, Jean Pennycook, Gerald L. Kooyman, Kara Schmidlin, Simona Kraberger, David G. Ainley, Arvind Varsani

**Affiliations:** 1 Oregon Cooperative Fish and Wildlife Research Unit, Department of Fisheries and Wildlife, Oregon State University, 104 Nash Hall, Corvallis, OR 97331, USA; 2 US Geological Survey, Oregon Cooperative Fish and Wildlife Research Unit, Department of Fisheries and Wildlife, Oregon State University, 104 Nash Hall, Corvallis, OR 97331, USA; katie.dugger@oregonstate.edu; 3 Point Blue Conservation Science, Petaluma, CA 94954, USA; gballard@pointblue.org (G.B.); melrod@pointblue.org (M.E.); aschmidt@pointblue.org (A.S.); alescroel-RA@pointblue.org (A.L.); djongsomjit@pointblue.org (D.J.); 4 Laboratório de Patologia Comparada de Animais Selvagens (LAPCOM), Departamento de Patologia, Faculdade de Medicina Veterinária e Zootecnia, Universidade de São Paulo, São Paulo 05508-060, Brazil; vruoppolo@gmail.com; 5 School of Environmental Sciences, Institute for Land, Water and Society, Charles Sturt University, Albury 2678, Australia; mmassaro@csu.edu.au; 6 HT Harvey and Associates, Los Gatos, CA 95032, USA; jean.pennycook@gmail.com (J.P.); dainley@penguinscience.com (D.G.A.); 7 Scholander Hall, Scripps Institution of Oceanography, University of California, La Jolla, San Diego, CA 92093-0204, USA; gkooyman@ucsd.edu; 8 The Biodesign Center for Fundamental and Applied Microbiomics, Center for Evolution and Medicine, School of Life sciences, Arizona State University, Tempe, AZ 85287-5001, USA; Kara.Schmidlin@asu.edu (K.S.); simona.kraberger@asu.edu (S.K.); 9 Structural Biology Research Unit, Department of Integrative Biomedical Sciences, University of Cape Town, Observatory, Cape Town 7701, South Africa

**Keywords:** *Pygoscelis adeliae*, *Circoviridae*, Antarctica, Ross Island, Cape Crozier

## Abstract

Understanding the causes of disease in Antarctic wildlife is crucial, as many of these species are already threatened by environmental changes brought about by climate change. In recent years, Antarctic penguins have been showing signs of an unknown pathology: a feather disorder characterised by missing feathers, resulting in exposed skin. During the 2018–2019 austral summer breeding season at Cape Crozier colony on Ross Island, Antarctica, we observed for the first time an Adélie penguin chick missing down over most of its body. A guano sample was collected from the nest of the featherless chick, and using high-throughput sequencing, we identified a novel circovirus. Using abutting primers, we amplified the full genome, which we cloned and Sanger-sequenced to determine the complete genome of the circovirus. The Adélie penguin guano-associated circovirus genome shares <67% genome-wide nucleotide identity with other circoviruses, representing a new species of circovirus; therefore, we named it penguin circovirus (PenCV). Using the same primer pair, we screened 25 previously collected cloacal swabs taken at Cape Crozier from known-age adult Adélie penguins during the 2014–2015 season, displaying no clinical signs of feather-loss disorder. Three of the 25 samples (12%) were positive for a PenCV, whose genome shared >99% pairwise identity with the one identified in 2018–2019. This is the first report of a circovirus associated with a penguin species. This circovirus could be an etiological agent of the feather-loss disorder in Antarctic penguins.

## 1. Introduction

**Figure 1:**
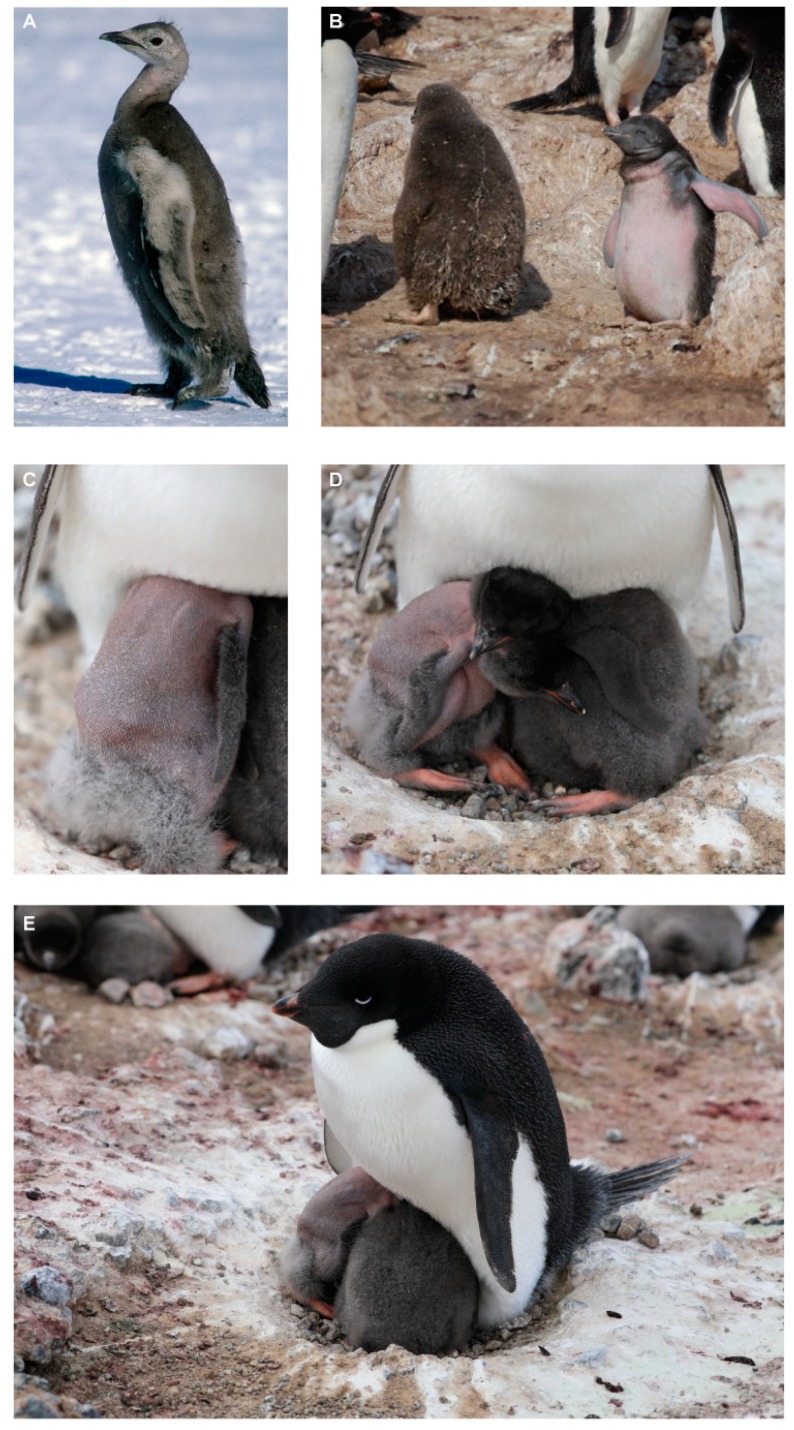
(**A**) Emperor penguin chick with feather-loss disorder (1996) at Cape Washington, northern Victoria Land, and in southern Victoria Land, Ross Island; (**B**) Adélie penguin chick with feather-loss disorder at Cape Royds, southern Victoria Land (2011–2012 breeding season); (**C**) Adélie penguin chick with feather-loss disorder at Cape Crozier (2018–2019 breeding season); (**D**) Adélie penguin chick affected by feather-loss disorder (2018–2019 breeding season) on a nest at Cape Crozier with fully feathered sibling; and (**E**) an asymptomatic parent.

A feather condition of unknown origin has been documented affecting the chicks of several penguin species during the past couple of decades. At Cape Washington, northern Victoria Land coast, in 1996, we observed an Emperor penguin (*Aptenodytes forsteri*) chick with feather disorder (Figure 1A). This feather disorder of the affected chick appeared to be visually similar to a disorder observed in African penguin (*Spheniscus demersus*) chicks from a South African rehabilitation centre in 2006 and in non-captive animals in 2008 [1]. Around the same time, penguin chicks with a similar condition were identified in wild Magellanic penguins (*Spheniscus magellanicus*) in four colonies in Argentina [1]. The Magellanic penguin chicks lost their coats of down and remained featherless until the growth of their normal juvenile plumage. Some of the chicks were able to survive until their adult plumage grew normally; however, this was largely dependent on the severity of the disorder and the weather conditions (temperature and wind speed). The Magellanic penguin chicks that survived grew more slowly and were smaller at fledgling age than feathered chicks [1]. Without immediate regrowth of feathers in these *Spheniscus* spp., it is thought that energy usually utilized for body growth is instead used for thermoregulation and metabolism. In the 2011–2012 austral summer breeding season, we observed an Adélie penguin (*Pygoscelis adeliae*) chick with the feather disorder at Cape Royds (Figure 1B), but it grew its adult feathers and fledged, appearing healthy. In the 2013–2014 breeding season, premature loss of feathers resulting in exposed bare skin was noted by Barbosa et al. [2] in Adélie penguins at the Esperanza Bay colony (Hope Bay, Antarctic Peninsula).

During the 2018–2019 breeding season, we observed an Adélie penguin chick missing most of the down on its body at the Cape Crozier colony on Ross Island, located ~130 km (along the coast) from Cape Royds. The Cape Crozier Adélie penguin colony is one of the largest in the world [3,4] and it is the largest within a four-colony metapopulation associated with Ross Island. During routine monitoring of the penguin colony, the ~10-day-old chick was found with only a few patches of down on its head, flippers, and lower body, while the rest of the body was bare and showing partial feather shafts without any lesions or scabs observed on the skin (Figure 1C). This chick’s sibling of similar size appeared normal and fully feathered with the typical down of a chick at this age (Figure 1D), and the parent appeared normal (Figure 1E). The appearance of the “diseased” chick was very similar to that observed earlier for Adélie penguins at Cape Royds (Figure 1B) and by Barbosa et al. [2] at Esperanza Bay.

It has generally been hypothesized that the disorder in penguins that results in missing feathers is due to a viral etiological agent [2,5]. While investigating a feather disorder condition in adult Adélie penguins (small patches of missing feathers; incidence of 1 in 1000 animals observed), Grimaldi et al. [5] identified an astrovirus-like sequence by using a metagenomic approach on pooled blood samples from affected birds. Various other viruses have also been identified as infecting Antarctic penguins, reviewed in Smeele et al. [6]. However, no viral agent has been identified that could result in such disease manifestation in penguins. In other avian species, especially parrots, the disorder resulting in feather abnormalities has been attributed to circoviruses (family *Circoviridae*), whereas “French moult” in which there is impairment of primary flight feather development, is a result of polyomaviruses (family *Polyomaviridae*) [7,8]. Previously, our research group has identified a polyomavirus in Adélie penguin faecal samples (2012–2013 breeding season) from the Cape Royds colony on Ross Island [9], but no animal was observed that season with any obvious feather disorder.

Here, we report the identification of a novel circovirus associated with Adélie penguins. Circoviruses (family *Circoviridae*) have single-stranded circular DNA genomes (~1.8–2.2 kb) and are classified into two genera, *Circovirus* and *Cyclovirus* [10]. They encode two main proteins, the capsid protein (CP) on the complementary sense strand and the replication-associated protein (Rep) on the virion sense strand. Circoviruses infect a diverse range of avian, fish and mammalian species. The best-characterized circoviruses are those that infect pigs (porcine circovirus type 2 (PCV2)) and those that affect birds in the order Psittaciformes (beak and feather disease virus (BFDV)) [11,12,13]. PCV2 has been implicated in a number of porcine diseases [14], most notably postweaning multisystemic wasting syndrome (PWMS), whereas BFDV causes psittacine beak and feather disease (PBFD) in parrots (present as a peracute, acute, or chronic disease). Neonates to fledglings tend to suffer from the peracute and acute forms of PBFD, in which sudden death occurs with no (peracute) or mild (acute) feather dystrophy [15,16]. The chronic form of PBFD is more prevalent in older psittacine birds where symptoms begin with depression, diarrhea, lethargy, and feather deformities. Feather deformities include constricted or necrotic feather shafts, curled or clubbed feathers, hemorrhage into the feather shaft and retained feather sheaths [17,18,19,20,21]. In severe cases of PBFD, especially in cockatoos, beak and claw deformities can occur [11,17,20]. BFDV is highly contagious, and the virus can be spread horizontally through shedding in crop secretions, feather dust and feces [22], and vertically from infected hens to embryonated eggs [23].

Feather abnormalities and syndromes similar to those of PBFD have been noted for other avian circovirus infections, including duck circovirus (DuCV) [24,25], canary circovirus (CanCV) [26] and finch circovirus (FiCV) [27,28,29]. In Senegal doves, a feather abnormality has been identified and attributed to circovirus [30,31], which was serologically determined to be different from BFDV, but its genome has not been determined. Circoviruses affecting other avian species do not appear to cause feather dystrophy or loss; however, they do appear to be lymphotropic and therefore are also assumed to be immunosuppressive [8,32].

## 2. Materials and Methods

The fieldwork was conducted under the supervision of Oregon State University’s Institutional Animal Care and Use Committee Corvallis, OR, U.S.A. and Antarctic Conservation Act Permit #2006-010 from the U.S. National Science Foundation (NSF).

A physical examination of the Crozier Adélie penguin chick missing most of the down on its body (Figure 1C,D) was not possible at the time of the first observation, and four days later it was missing from the nest. The downy sibling was still present, guarded by a parent. Three days after the disappearance of the affected chick, the nest site was vacant, and a sample of guano was collected from the nest into a 1.5 mL tube and stored/transported at ~4 °C. Viral DNA was extracted from the guano as previously described for Antarctic samples handled by our team [9,33,34]. Briefly, ~1 g of guano was resuspended in 1 mL of SM buffer (0.1 M NaCl, 50 mM Tris/HCl at pH 7.4, 10 mM MgSO_4_) and homogenized. The homogenate was centrifuged at 10000× *g* to pellet debris, and the supernatant was sequentially filtered through 0.45 μm and 0.2 μm syringe filters. Viral DNA was extracted using 200 μL of the filtered solution using the High Pure Viral Nucleic Acid Kit (Roche Diagnostics, Indianapolis, IN, USA). Viral circular DNA was enriched using the TempliPhi™ kit (GE Healthcare, Chicago, IL, USA) by rolling circle amplification (RCA). The RCA products were used to prepare Illumina libraries using the Nextera DNA Flex Library Prep Kit and sequenced on an Illumina4000 sequencer (2 × 100 bp library). The resulting paired-end reads were de novo assembled with metaSPAdes v 3.12.0 [35]. Contigs >500 nts were screened for viral-like sequences using BLASTx [36] against a viral protein NCBI RefSeq database (downloaded on 10 July 2019). One contig (~1900 nts) was identified as having similarities to circovirus capsid protein (CP) and replication-associated protein (Rep). A pair of abutting primers in the capsid protein (*cp*) gene region was designed (CP-31824-F 5′-TGTTTCTTTCGATAGGTTGAATTCAAATTGTCGTT-3′; CP-31824-R 5′-ACACCAGGTCAGTTAGAGTTTGATAGTGATTATCT-3′) based on the contig to recover the complete genome of the virus. The genome was amplified using the primer pair and 0.5 μL of the RCA product as a template with KAPA HiFi HotStart DNA Polymerase (Kapa Biosystems, Wilmington, MA, USA). The amplicon was resolved on a 0.7% agarose gel, gel-purified, cloned into pJET1.2 plasmid (ThermoFisher, Waltham, MA, USA) and Sanger-sequenced at Macrogen Inc. (Seoul, Korea). The Sanger-sequenced genome (1988 nts) was assembled from Sanger-sequenced contigs using Geneious V11 [37]. To verify that we had recovered the full genome and were not missing any bases around the 5′ ends of the abutting primer pair, a second pair of abutting primers, CP-31824-F and CP-31824-R, were designed for the *rep* gene (Rep-56233-F 5′-TAACCATCCCACCAGTCACCTTTC-3′; Rep-56233-F 5′-CCACGAAGATATAGTCATCATAGA-3′). The same protocol as above was followed to amplify, clone and sequence the full genome. Both cloned genomes were found to be 100% identical.

**Figure 2:**
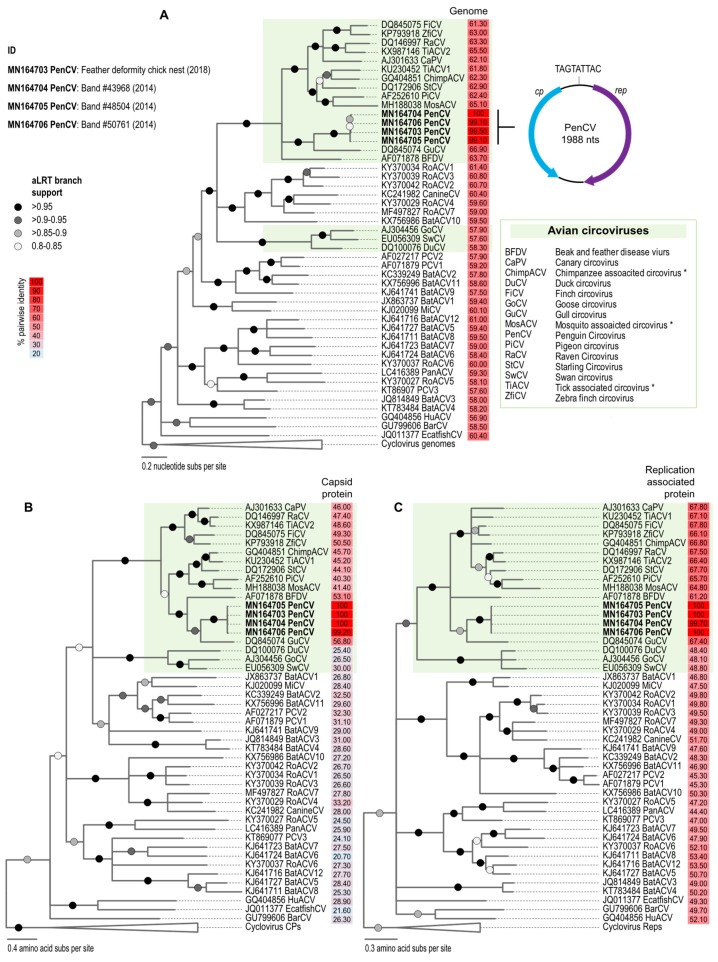
(**A**) A maximum likelihood phylogenetic tree of the aligned genome sequences of circoviruses and an illustration of the genome organization of penguin circovirus (PenCV); (**B**) a maximum likelihood phylogenetic tree of the capsid protein (CP) amino acid sequences; and (**C**) replication-associated protein (Rep) amino acid sequences. The percentage pairwise identities relative to PenCVs are shown next to the taxa names (with GenBank accession numbers). * Viruses likely infect avian species, as they have been recovered from fecal and insect vectors.

To identify the circovirus in previous samples, we tested 25 cloacal swabs that had been collected (in the 2014–2015 Adélie penguin breeding season) from adult birds ranging from 7 to 18 years of age (8 females and 17 males) and stored in 1000 µL of the UTM™ Viral Transport Media (Copan, U.S.A.). Viral DNA was extracted directly from 200 μL of each of the transport media as described above, and circular molecules were amplified using RCA. We tested these 25 samples with the primer pair CP-31824-F/CP-31824-R and recovered complete genomes of the circoviruses from three samples, with genome sizes of 1986–1988 nts (Figure 2A).

The genomes of representative circoviruses and their Rep and CP amino acid sequences, together with those from this study, were aligned separately using MUSCLE [38]. The full genome and CP and Rep sequence alignments were used to infer maximum likelihood phylogenetic trees using PhyML [39]. A GTR+I+G nucleotide substitution model was identified as the best fit model determined using jModelTest [40] for the full genome alignment, whereas an rtRev+G+I amino acid substitution model was determined as best fit using ProtTest [41] for the Rep and CP sequence alignments. The phylogenetic trees were rooted with sequences of cyloviruses and branches with <0.8 aLRT [42] branch support collapsed using TreeGraph2 [43].

## 3. Results and Discussion

The genomes of the four PenCVs (GenBank accession numbers MN164703–MN164706) share >99% genome-wide identity. In the genome, we identified the replication-associated protein gene (*rep*; 870 nts) on the virion sense and the capsid protein gene (*cp*; 727 nts) on the complementary strand. We also identified the conserved nonanucleotide motif TAGTATTAC (Figure 2A). Pairwise comparisons of the representative genomes from each species of the genus *Circovirus* (downloaded 12 June 2019) using SDT v1.2 [44] revealed that the Adélie penguin guano-associated circovirus genome shares <67% genome-wide nucleotide identity with other circoviruses. Thus, this represents a new species of circovirus, which we named penguin circovirus (PenCV), based on the classification criterion (species demarcation threshold for circoviruses is 80% genome-wide pairwise identity) established for the family *Circoviridae* [10].

The PenCV sequences are most closely related to those of gull (*Larus* spp.) circoviruses (GuCV), sharing ~67% genome-wide identity, and form a well-supported cluster in the maximum likelihood phylogenetic tree (Figure 2A). A similar clustering is observed for the CP and Rep amino acid sequence-based phylogenies with the CP of PenCV, sharing ~57% identity, whereas the Rep shares ~67% identity with that of GuCV. PenCV, more broadly, clusters with the avian circoviruses identified in canary (CaPV), finch (FiPV), parrot (BFDV), pigeon (PiCV), raven (RaCV) and starling (StCV), sharing 61%–67% identity, whereas their Reps and CPs share 48%–68% and 25%–57% identity, respectively (Figure 2).

The penguins whose cloacal swabs, collected in the 2014–2015 season, were found to be circovirus-positive had a known breeding history that included the following in 2014–2015: (1) a penguin 15 years old, who successfully raised at least one chick to crèche but was not resighted since 2014–2015; (2) a penguin 10 years old, who successfully raised at least one chick to crèche and was resighted as a breeder every year since; and (3) a penguin 8 years old, who successfully raised one chick to crèche and was resighted every year since. This indicates that the PenCV was present in the Adélie penguins in the 2014–2015 austral summer with no clinical signs of feather-loss disorder but a viral prevalence of 12% (3 out 25 tested were PenCV-positive). In avian species, circovirus infections are subclinical or mild [45], and clinical signs depend on how fast the disease progresses [8]. In the case of beak and feather disease virus (BFDV) of parrots, the best studied of avian circoviruses, the virus is endemic with a high seroprevalence of antibodies (indicative of active infection or prior exposure post-viral clearing); active infection prevalence in sampled animals ranges from 15% to 28%, though there are some instances where it is >50% [17,46,47,48,49,50].

## 4. Conclusions

Since 1996, surveys have been conducted every two to seven days from November to January of each year to monitor banded birds at the three main colonies of Adélie penguins in the Ross–Beaufort metapopulation (Capes Crozier, Bird, and Royds [51,52]). However, sporadic surveys have occurred since the 1960s [53]. A feather disorder resulting in most of the down missing in Adélie penguin chicks had not been observed in Antarctic penguins until the 2011–2012 breeding season at Cape Royds and later at Esperanza Bay, Antarctic Peninsula, in 2014. It is not likely that penguins from these two colonies ever associate, as they are separated by over 5000 km. Our findings at Cape Crozier add another colony where this disorder has been recorded in recent years.

In view of our results, it is possible that PenCV is common on Ross Island amongst Adélie penguins with no clinical signs of the infection. Accordingly, we cannot rule out the possibility that the feather disorder is a result of a combination of disorders ranging from genetic to viral and other microbial co-infections. To investigate the epidemiology and associated disease outcomes, a larger survey is needed. It would incorporate variable spatial and temporal scales among Antarctic penguin colonies in order to determine the rates of the PenCV infection. Further, this would help determine whether virus presence is correlated with disease or whether the disease outcome is only an extreme case and a result of other compounding factors such as co-infections, genetic disorders, immune suppression or abiotic factors.
